# Belief That Caffeine Ingestion Improves Performance in a 6-Minute Time Trial Test without Affecting Pacing Strategy

**DOI:** 10.3390/nu16020327

**Published:** 2024-01-22

**Authors:** Fernando Valero, Fernando González-Mohíno, Juan José Salinero

**Affiliations:** 1 Sport Training Lab, Faculty of Sport Sciences, University of Castilla-La Mancha, 45071 Toledo, Spain; fernando.valero1@alu.uclm.es (F.V.); fernando.gmayoralas@uclm.es (F.G.-M.); 2 Facultad de Ciencias de la Vida y de la Naturaleza, Universidad Nebrija, 28248 Madrid, Spain

**Keywords:** placebo effect, expectancy, ergogenic aids, running performance

## Abstract

This study aimed to analyze the placebo effect associated with caffeine ingestion on running performance. Methods: Thirteen recreationally trained males in long-distance running (age: 38.5 ± 11.9 years, 11.0 ± 8.8 years of running experience) performed a 6 min time trial test in each experimental condition (caffeine-informed/placebo ingested (placebo) and non-ingested (control)) separated by 7 days. The total distance covered was measured, and partial times of each 400 m were used to analyze the pacing strategy. Heart rate and kinematic variables were recorded for each split. In addition, the rate of perceived exertion and prevalence of caffeine side effects was measured using questionnaires. Results: Placebo ingestion improved running performance in the 6 min time trial test (1668 ± 139 m placebo vs. 1642 ± 158 m control, t = 2.49; *p* = 0.03; moderate ES = 0.69), while pacing, heart rate, RPE, and kinematic variables were similar between conditions (*p* > 0.05 in all cases). Minor side effects were reported. Conclusions: Beliefs of caffeine ingestion can improve running performance at speeds around maximal aerobic speed in recreationally trained runners without affecting pacing strategy so this “nutritional” strategy could be useful in competition scenarios. As a practical application, recreationally trained runners could improve ≈5 s in 1500 m or mile competitions.

## 1. Introduction

The placebo effect (i.e., an improvement derived from something that has no effect by itself) has been broadly studied in medicine and psychology in various clinical conditions [1]. In recent decades, an increasing interest has emerged in sports sciences research, where previous reviews have found small to moderate exercise performance improvements [2,3]. The possibility of improving sports performance, with the simple placebo effect, has led to this effect being studied in recent years in the sports field from different perspectives, such as nutritional or mechanical ergogenic aids [3].

Most studies on the placebo effect on sports domains have been carried out from the perspective of ergogenic aids, such as carbohydrates [4], amino acids [5], or sodium bicarbonate [6]. Nevertheless, the main source of placebo research in sports performance has been associated with caffeine [3], probably due to the well-known ergogenic effects of this substance on sports performance [7,8,9]. Since exercise performance can be influenced not only by the pharmacological effects of the ergogenic aid but also by the expectancy effects caused by beliefs about the efficacy of such substance [3,6,10], caffeine’s reputation makes it an ideal candidate to influence the athlete’s expectancies. In fact, three out of four elite athletes (data from urine samples obtained in official competitions for anti-doping analysis) consume caffeine during competition periods, and the use of caffeine in sports increased from 2008 to 2015 [11].

Athletes of individual sports or athletes of sports with an aerobic-like nature (i.e., rowing, cycling, triathlon, etc.) are more prone to using caffeine in competition [11]. Although nowadays there is solid evidence about the ergogenic effect of caffeine on strength performance [8,12,13], there were controversial results on this topic years ago [14]. In contrast, the ergogenic properties of caffeine on endurance performance have been well-established for a long time [9,15,16,17], and athletes in these sports could be more prone to generate expectancies about its positive effects. So, while a few placebo–caffeine-related research studies in the sports context have been focused on strength performance [5,18,19,20], most studies analyzed this topic on endurance performance [21,22,23,24,25,26].

Although there has been an increasing interest in recent years, research about this topic is still scarce. The use of placebos to improve running performance has been analyzed by a few studies [25,26]. Hurst, Schiphof-Godart, Hettinga, Roelands, and Beedie [26] analyzed the placebo effect on pacing and running performance in 11 well-trained middle-distance athletes. They performed a 1 km time trial (TT) test under four conditions (caffeine informed/caffeine ingested, caffeine informed/placebo ingested, placebo informed/caffeine ingested, and placebo informed/placebo ingested). Running performance improved in the two conditions where runners were informed about caffeine ingestion (caffeine informed/caffeine ingested, and caffeine informed/placebo ingested), without differences in RPE. In addition, in both cases, the improved performance was associated with an increased pace during the first half of the trial, indicating that the reported caffeine/ingested placebo had some influence on a psychological level, and they believed that he had actually ingested caffeine. In the same way, in the study of Rohloff, Souza, Ruiz-Moreno, Del Coso, and Polito [25], 22 recreational runners performed a 4 km TT test under three conditions (control, caffeine informed/placebo ingested, and caffeine informed/caffeine ingested). The placebo similarly improved running performance to caffeine ingestion, without differences between treatments for RPE. Therefore, caffeine-induced expectancy may be one of the mechanisms behind the ergogenic effect of this stimulant on endurance exercise.

These studies found similar improvements with placebos as with an ergogenic aid such as caffeine [25,26]; therefore, it seems that the placebo effect could be incorporated as a simple strategy to improve performance since it does not present any risk to the health of athletes, diminishing the possible side effects of other ergogenic aids used by athletes, such as caffeine, where it has been shown that it can cause nervousness, gastrointestinal discomfort, or insomnia [27,28]. In addition, higher doses of caffeine (i.e., 9 mg/kg of body mass) drastically increased the frequency of the adverse side effects in comparison with moderate doses (i.e., 3 to 6 mg/kg). For example, with this higher dose, 38% of participants reported gastrointestinal problems, and 54% of subjects reported insomnia or sleep disturbances [28]. Insomnia or altered sleep-wake cycles could negatively affect sports performance, so the use of caffeine, especially in competitions in the afternoon, could interfere with recovery and sports performance in successive competition days (e.g., heats, semi-finals and final in athletics).

Nevertheless, given the scarce scientific literature on this topic, further investigations are necessary to consolidate these outcomes. This study aimed to analyze the placebo effect associated with caffeine ingestion on pacing strategy and running performance during a 6 min TT test.

## 2. Materials and Methods

### 2.1. Subjects

An a priori sample size estimation revealed that at least 9 participants were required to investigate the potential placebo effect of caffeine with an effect size of 1.15 tested with a two-tailed paired sample *t*-test (1 − β = 0.8; α = 0.05). This calculation was based on the effect size obtained with placebo vs. control conditions of Hurst et al.’s investigation [26], and it was performed with G*Power (v3.1.9.7) software. A call for participants was performed in local athletics track clubs. Thirteen recreationally trained males in long-distance running (mean ± SD, 38.5 ± 11.9 (range 20–59) years old; 72.2 ± 6.4 (63–85) kg; 175.5 ± 6.5 (166–168) cm; 11.0 ± 8.8 (1–30) years of running experience, 46.9 ± 17.1 (25–85) km of weekly training volume) volunteered to participate in this study. The inclusion criterion was that they performed running training regularly for at least 3 days/week the last year, ensuring that they were sufficiently experienced in race pace control and were adults (i.e., ≥18 years old). All of them had no physical limitations or musculoskeletal injuries that could affect running performance. Before the study, all participants were informed about the testing protocols and possible risks involved and were invited to provide written informed consent. The study was performed in accordance with the principles of the Declaration of Helsinki, and the experimental protocols were approved by the local ethics committee (ref. 28.1.2021CEI-UCJC; Comité de Ética de la Investigación de la Universidad Camilo José Cela, 2 February 2021).

### 2.2. Design

A repeated, randomized, and counterbalanced experimental design was used to compare the effects of the ingestion of a placebo reported as caffeine (placebo) and a control situation where no substance was ingested (control), in which each subject performed in two 6 min time trial tests (separated by 7 days). Seven participants started with the control condition, and 6 participants started with the placebo condition.

### 2.3. Procedure

In two sessions separated by 7 days, participants were instructed to avoid previous caffeine ingestion and not perform heavy exercise in the 48 h prior. Twenty-four hours before each experimental trial, participants adopted a similar sleep pattern, diet, and fluid intake regimen. They performed both tests at the same time of day to avoid the effects of circadian rhythms with similar weather conditions (rainless and similar ambient temperature). Participants performed a 6 min TT test in each ingestion condition, attempting to maintain the highest constant speed during the test to cover the longest distance possible. This test was validated as a predictor of maximal aerobic speed in endurance athletes [29,30]. The participants were familiarized with this test in their usual training regimen, except in two cases, for which a pre-session was conducted 3 weeks before the experimental sessions. Before the test started, participants were informed about the ergogenic properties of caffeine, and how previous studies had found performance improvements on similar running tests to this 6 min TT test (similar distance and/or duration). After this, participants performed a standardized warm-up that consisted of 15 min of continuous running, mobility exercises, running drills (“A” skips: skip with high knees, run with high knees, “B” skips, and butt kicks; performing 2 × 15 m repetitions each), and 2 × 150 m progressive runs. After that, participants rested for 5 min before the 6 min TT test started. In this interval, participants were instructed to cover the longest possible distance at a constant speed (maximum effort) on a standard 400 m outdoor synthetic athletics track (with a homogeneous curve radius of 36.5 m), according to the World Athletics’ standards (Mondo, Italy). The total distance covered in the 6 min TT test was also determined (m). In each of the 6 min TT tests, the partial times for each 400 m were used to analyze the pacing strategy. In addition, heart rate was recorded with an H9 pectoral band connected to a Polar PacerPro watch (Polar Electro Oy, Kempele, Finland). The main kinematic variables of the gait cycle (step frequency, vertical oscillation, and contact time) were measured for each 400 m split using a Stryd power meter device (Stryd, Boulder, CO, USA), sampling at 1000 Hz. This device used a triaxial accelerometer that was reliable and valid for these measures [31] and was placed on the right shoelaces of the runners. The data were analyzed using the Stryd Power Center program available on the web (https://www.stryd.com/powercenter/, accessed on 1 December 2023). For each 6 min TT test, participants were instructed to wear the same shoe models (visually checked by the researchers) and similar clothes in both tests.

The participants, after being informed about the ergogenic properties of caffeine, were given a piece of gum (1.4 g, Gumlink Confectionery Company, Vejle, Denmark) with supposed caffeine, which they had to chew for 5 min during the mobility exercises in the warm-up. They were informed that the gum allowed the absorption of an optimal dose to improve running performance. These chewing gums did not actually contain any amount of caffeine or any other psychoactive substance. The piece of gum was administered in an unidentifiable plastic bottle to avoid identifying the gum brand.

The participants did not receive any information about split times, heart rate, or kinematic variables. The clock was blinded with a tape on the screen.

The morning after carrying out the tests, they were sent a link to fill out an electronic form on possible side effects (Google Forms), where they had to indicate on a dichotomous scale (i.e., yes/no) the presence or not of each of the possible side effects, such as nervousness, digestive problems, or difficulty sleeping. This questionnaire was previously used to assess the side effects derived from caffeine ingestion in the sport domain [27].

### 2.4. Statistical Analysis

The results of each test were entered into the statistical package SPSS v 28.0 (IBM Corp., Armonk, NY, USA). The normality of each variable was initially tested with the Shapiro–Wilk test. Except for heart rate, all variables were not different compared to the normal distribution. A *t*-test for paired samples was used to analyze the differences between the placebo condition and the condition without intake in the global variables, Student t-test for variables with a normal distribution, and Wilcoxon test for variables (heart rate) without a normal distribution. A two-factor repeated measures ANOVA (partial × condition) was used for the variables measured during each 400 m split. We included 400, 800, 1200, and up to 6 min (i.e., any distance above 1200) distance splits to unify the runners. Additionally, the sphericity assumption was checked with Mauchly’s test. If this assumption presented a probability of *p* < 0.05, the Greenhouse–Geisser correction was used. Data are presented as mean ± standard deviation. In all statistical tests, a level of *p* < 0.05 was set to establish statistically significant differences. In addition, the effect size (ES) for the condition was calculated using Cohen’s d, interpreting it as <0.20 trivial, ≥0.20–0.59 small, ≥0.60–1.19 moderate, ≥1.20–1.99 large, and ≥2.00 very large according to the recommendations of Hopkins [32].

## 3. Results

**Figure 1:**
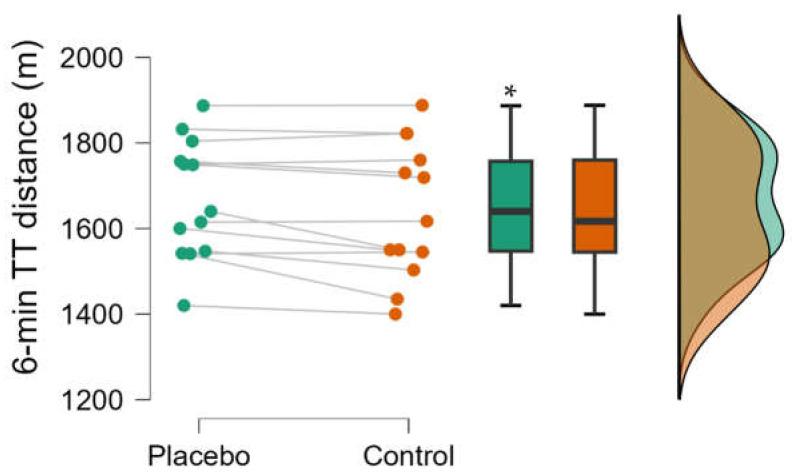
Total distance covered in the 6 min time trial test. Individual values, box-plot, and distribution of 13 recreational runners in the placebo (green) and the control condition (orange). * Significant difference between the placebo and the control condition (*p* < 0.05).

Figure 1 depicts the total distance covered in the 6 min TT test in the experimental conditions (placebo and control). Belief in caffeine ingestion improved overall performance in the test (1668 ± 139 m in the placebo condition vs. 1642 ± 158 m in the control condition, t = 2.49, *p* = 0.03; moderate ES = 0.69). Individual analysis showed that 8 out of 13 participants improved running performance in the placebo conditions, while the other 5 did not.

**Figure 2:**
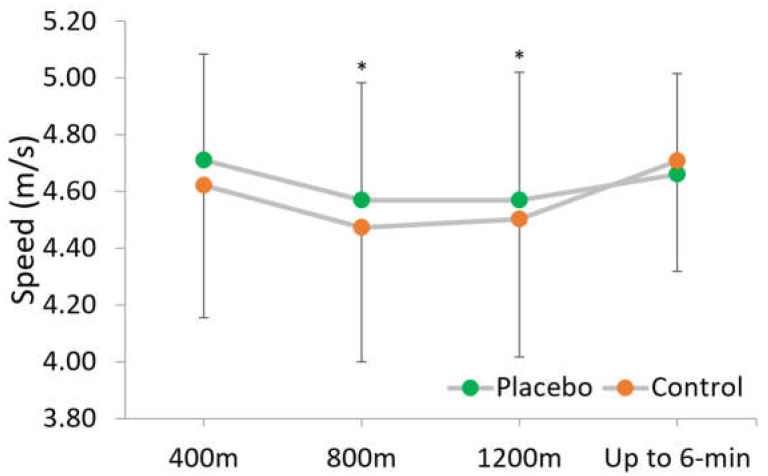
Speed at each split in the 6 min time trial test. * Significant difference between the placebo and the control condition (*p* < 0.05).

The split analysis (Figure 2) did not reveal an interaction between condition and pacing (F = 2.85; *p* = 0.09; ƞ^2^partial = 0.19), nor an effect for condition (F = 3.81; *p* = 0.08; ƞ^2^partial = 0.24). There was a significant main effect for split (F = 4.39; *p* = 0.03; ƞ^2^partial = 0.27). In both conditions, the pacing was U-shaped, with a faster start and finish. Pairwise comparisons showed greater speed in the placebo condition in the second (4.57 ± 0.41 m/s with placebo vs. 4.47 ± 0.47 m/s in control condition; F = 5.77; *p* = 0.03; moderate ES = 0.67) and third split (4.57 ± 0.45 m/s with placebo vs. 4.50 ± 0.49 m/s in control condition; F = 4.90; *p* = 0.04; moderate ES = 0.61).

Heart rate (Table 1) was similar between conditions (F = 1.47; *p* = 0.25; ƞ^2^partial = 0.13), with no significant interaction effect between condition and split (F = 0.37; *p* = 0.65; ƞ^2^partial = 0.04). Logically, heart rate increased progressively in each split (F = 373.9; *p* < 0.01; ƞ^2^partial = 0.97). Effect sizes in pairwise comparisons were small in all splits (ES = 0.22 to 0.40) and in the overall test (ES = 0.45). The rate of perceived exertion was too similar between conditions (t = 0.90; *p* = 0.39, small ES = 0.25). In addition, regarding the kinematic variables, there were no significant interactions between the condition and split in any variable (*p* > 0.05 in all cases) nor a significant main effect for conditions with trivial to small effect sizes (*p* > 0.05 in all cases).

Finally, reported side-effects were similar between both conditions, with scarce positive responses to increased nervousness (n = 1; 7.7%), muscular pain (n = 1; 7.7%), gastrointestinal discomfort (n = 1; 7.7%), increase in urine production (n = 1; 7.7%), or headache (n = 2; 15.4%) after placebo ingestion. Three out of thirteen runners (23.1%) reported increased activeness after placebo ingestion. In the control condition (i.e., no substance was administered), runners reported no side effects, except one runner (7.7%) who reported increased activeness in this control condition. None of the runners reported sleeping problems.

## 4. Discussion

The aim of this study was to analyze the placebo effect associated with caffeine ingestion on pacing strategy and running performance during a 6 min TT test. The main finding was that belief of caffeine ingestion produced a greater distance covered (+1.61%; moderate ES = 0.69) in the placebo condition compared to the control condition, without influence on pacing strategy, heart rate, rate of perceived exertion, or kinematics variables.

Beliefs of caffeine ingestion produced a mean increase of 46 m (1.61%) in the 6 min TT test from 1642 ± 158 m in the control condition to 1668 ± 139 m in the placebo condition. Thus, the speed in the placebo condition was 4.63 ± 0.39 m/s (i.e., 16.7 km/h) while in the control condition was 4.56 ± 0.44 m/s (i.e., 16.4 km/h). The 6 min TT is a common test used to evaluate maximal aerobic speed in runners [30]. Although there is great inter-individual variability, 6 min is an average value of the time limit to maintain this maximal aerobic speed [29,33,34]. Therefore, maximal aerobic speed could be significantly improved with the effect of believing to have ingested caffeine. In the same way, in this running level, this performance improvement could be extrapolated to middle-distance races such as 1500 or mile competitions, and so, the belief of caffeine ingestion could improve their performance in these distances by ≈5 s. Previous studies in highly trained runners found similar improvements in 1000 m time trials (≈3 min), where runners improved by 1.9% (175.9 ± 0.6 s in control condition vs. 172.6 ± 0.6 s after placebo ingestion) when they were told they ingested caffeine but actually ingested a placebo [26]. They increased running speed from 5.69 m/s (i.e., 20.47 km/h) to 5.80 m/s (i.e., 20.86 km/h). Therefore, according to our outcomes and Hurst et al. [26], the placebo effect could improve running performance both in recreational and trained runners in middle-distance competitions or tests between 3 to 6 min.

This placebo effect associated with caffeine has been found in other endurance sports, such as cycling, where the belief of caffeine ingestion improved different time trial tests [22,23]. Indeed, a dose–response relationship was found in a 10 km TT test, with cyclists reducing performance by 1.4% when they believed they had ingested a placebo and increasing performance by 1.3% when they believed they had ingested 4.5 mg/kg caffeine and 3.1% when they believed they had ingested 9.0 mg/kg caffeine [22]. According to our data and other previous research, the placebo effect could improve endurance performance by around ≈2–3%, and this could be explained by a variety of factors, such as fatigue endurance, pain tolerance, and motivation [1].

Individual analysis showed that 8 out of 13 participants improved their running performance in the placebo conditions, while the other 5 did not. However, participants who experienced decreased performance with the placebo ranged from −1 to −18 m (mean ± sd: 7 ± 7 m), while participants who improved with the placebo ranged from +10 to +107 m (47 ± 34 m). As we previously suggested, some participants may be more likely to respond to placebo than others [3,26,35], influenced by, but not limited to, the context in which the treatment is administered, the person administering it, and psychological aspects of the runner (personality, beliefs, and intentions) [3,26]. In this line, similar to ergogenic aids, it seems that there are responders and non-responders, and so, we must consider which individuals we can use this strategy on to improve performance and which we need to consider other options. The runner must believe in the effectiveness of caffeine for improving performance. However, the runner may not fully benefit from it [3].

Regarding the pacing strategy employed by the runners in both conditions, they follow a U-shape pattern typical for middle-distance events [36]. There was no alteration in the pacing strategy related to the belief of caffeine ingestion during the 6 min TT test (Figure 2). Therefore, it seems that the placebo effect increases only the endurance performance in middle-distance events by improving absolute speed, but not a better distribution of effort. Previous research in trained runners found that the belief of receiving caffeine influenced the pacing strategy, with a faster start (i.e., first 400-m) in a 1000 m running time trial [26]. In contrast, in greater running distances (4 km) and recreationally trained runners, Rohloff, Souza, Ruiz-Moreno, Del Coso, and Polito [25] found progressively reduced pacing in the control condition, while the reduction in running performance was not seen in the placebo condition. Nevertheless, our data did not achieve significant interaction between splits and placebo–control conditions. However, in our data, pairwise comparisons showed faster intermediate splits (second and third laps) and, although not significantly different, a best mean time in the first split (4.71 ± 0.37 in the placebo condition vs. 4.62 ± 0.47 m/s in the control condition; F = 1.70; *p* = 0.11, small ES = 0.47). Therefore, these data are in line with the previous work of Hurst, Schipof-Godart, Hettinga, Roelands, and Beedie [26].

Kinematic variables and heart rate did not change between conditions. We hypothesized that an increase in speed in the placebo condition would produce changes in kinematic variables and increase heart rate. However, maybe the inter-individual variability led to the inability to reach statistical significance. In the same way, RPE did not change between placebo and control conditions. While previous research suggested that the placebo effect could reduce RPE on short-term resistance exercise to failure [20], our data are according to previous research on running performance [25,26]. In this scenario, where runners were instructed to complete the race at 100% of their capabilities, they could be expected to score maximum effort in both conditions. Therefore, an RPE reduction could be expected mainly in submaximal efforts and not at maximal effort, as in our study.

Finally, the use of caffeine has increased in recent years in the sports context [11], maybe because of its reported ergogenic effect [7,8,9]. However, the ingestion of caffeine or caffeinated products is typically accompanied by several side effects, including insomnia or nervousness, among others [27,28], so this could interfere with sports performance. In this case, the use of placebos could be a safer strategy. In this research, a minority of runners reported any side effects after placebo ingestion. Previous research about caffeine ingestion and side effects found that caffeine ingestion produced a higher prevalence of side effects than placebo, such as insomnia (31.2 caffeine vs. 10.4% placebo; *p* < 0.001), nervousness (13.2 caffeine vs. 0% placebo; *p* = 0.002) and activeness (16.9 caffeine vs. 3.9% placebo; *p* = 0.007) in athletes of several sports [27]. However, belief in caffeine ingestion in our runners promoted a lower prevalence of nervousness (7.7%), and no one reported insomnia. Curiously, increased activeness after placebo ingestion in these runners (23.1%) was higher than that obtained in this previous research with real caffeine ingestion. As a practical application, in those sports where several rounds/competitions in consecutive days, such as cycling (several daily stages during 3 weeks) or sprint and middle-distance running in athletics (heats, semi-finals and final in international competitions), the use of placebos could be an effective strategy for the first round, reserving caffeine intake for the last round, where the subsequent sleep quality is less relevant (i.e., competition finished).

The experimental design employed in this investigation presents some limitations that should be addressed to enhance the application of the results. First, we did not collect biological samples to confirm compliance with the recommendations to avoid caffeine or other psychoactive substances in each of the experimental scenarios. So, we could not confirm the absence of caffeine in serum or urine samples. Similarly, recommendations were made to replicate the conditions of rest and nutrition, but this was only confirmed verbally with the participants. Second, participants were recreationally trained (running speed ≈ 17 km/h in ≈1 mile), so we must not extrapolate our results to a higher level of performance. However, Hurst et al. [26] found similar results in a higher level of running performance (running speed ≈ 20 km/h in 1 km). Finally, all participants were male runners, so we could not assess the placebo effect on running performance on female athletes. There is conflicting evidence regarding the influence of sex on the occurrence and magnitude of placebo effects in clinical trials in medical studies [37], with scarce research in the sport performance domain. Therefore, more research is needed on this topic, where studies about the placebo effect on endurance running performance are scarce, and almost all of them are performed on male participants.

## 5. Conclusions

Beliefs of caffeine ingestion improve performance by 1.6% in a 6 min time trial test without affecting the pacing strategy in recreationally trained runners. As a practical application, recreationally trained runners could improve ≈5 s in 1500 m or mile competitions. Based on these findings, the use of a placebo in middle-distance running competitions could be a smart strategy to improve performance without the use of psychoactive substances with potential adverse side-effects such as insomnia. It could be useful when sleep disturbances can affect the next training or competition.

## Figures and Tables

**Table 1:** Heart rate, rate of perceived exertion and kinematic variables in the 6 min TT test. Data from each split and the overall test.

		Control	Placebo	Statistic	*p*	Cohen’s *d*
Heart rate (bpm)	400 m	147.6 ± 12.3	148.8 ± 14.0	0.54	0.48	0.22
	800 m	165.2 ± 12.1 ^†^	167.1 ± 12.9 ^†^	1.78	0.21	0.40
	1200 m	168.6 ± 12.0 ^†^	170.7 ± 13.1 ^†^	2.32	0.27	0.34
	Rest	171.8 ± 11.8 ^†^	173.2 ± 12.5 ^†^	0.98	0.35	0.30
	Overall	162.9 ± 12.1	165.0 ± 13.6	1.49	0.12	0.45
Rate of perceived exertion (a.u.)	Overall	9.2 ± 0.6	9.3 ± 0.5	0.90	0.39	0.25
Step frequency (spm)	400 m	181.8 ± 10.2	183.6 ± 8.5	1.49	0.25	0.37
	800 m	180.6 ± 9.8	182.4 ± 8.0	1.61	0.23	0.38
	1200 m	181.4 ± 9.9	182.6 ± 8.5	1.82	0.21	0.41
	Rest	183.6 ± 9.3	184.2 ± 8.7	0.58	0.47	0.23
	Overall	181.9 ± 9.8	183.1 ± 8.1	1.08	0.31	0.33
Vertical oscillation (cm)	400 m	8.30 ± 0.95	8.26 ± 0.95	0.10	0.76	0.10
	800 m	8.35 ± 1.04	8.26 ± 0.95	0.48	0.50	0.21
	1200 m	8.27 ± 1.07	8.21 ± 1.02	0.32	0.58	0.17
	Rest	8.11 ± 1.07	8.12 ± 1.05	0.03	0.88	0.05
	Overall	8.27 ± 1.02	8.21 ± 0.96	0.66	0.53	0.20
Contact time (ms)	400 m	170.6 ± 20.6	164.3 ± 14.4	4.27	0.07	0.62
	800 m	173.6 ± 18.9	169.3 ± 14.6	3.80	0.08	0.59
	1200 m	173.7 ± 17.2	170.7 ± 14.5	1.80	0.21	0.41
	Rest	170.1 ± 15.0	168.0 ± 13.2	1.24	0.29	0.34
	Overall	171.9 ± 18.4	168.7 ± 13.3	1.37	0.33	0.42

^†^ Significant difference with the previous split.

## Data Availability

The datasets used and/or analysed during the current study are available from the corresponding author upon reasonable request. The data are not publicly available due to being data from an ongoing project.
